# Conversion of 4-*N*,*N*-dimethylamino-4’-*N*’-methyl-stilbazolium tosylate (DAST) from a Simple Optical Material to a Versatile Optoelectronic Material

**DOI:** 10.1038/srep12269

**Published:** 2015-07-20

**Authors:** Xiangdong Xu, Ziqiang Sun, Kai Fan, Yadong Jiang, Rui Huang, Yuejiang Wen, Qiong He, Tianhong Ao

**Affiliations:** 1 State Key Laboratory of Electronic Thin Films and Integrated Devices, Ministry of Education Key Laboratory of Photoelectric Detection & Sensor Integration Technology, School of Optoelectronic Information, University of Electronic Science and Technology of China (UESTC), Chengdu 610054, P.R. China; 2 Cooperative Innovation Center of Terahertz Science, University of Electronic Science and Technology of China (UESTC), Chengdu 610054, P.R. China

## Abstract

4-*N*,*N*-dimethylamino-4’-*N*’-methyl-stilbazolium tosylate (DAST) is an important optical material, but its poor conductivity limits applications in devices. To tackle this problem, we designed, prepared, and systematically investigated novel binary composite films that are composed of two-dimensional (2D) DAST and 2D graphene. Results indicate that both electrical and optical properties of DAST can be significantly improved by graphene addition. The negative steric effects of big DAST molecules that greatly trouble *ex-situ* synthesis can be efficiently overcome by *in-situ* synthesis, thus leading to better film quality and higher physical properties. Consequently, the *in-situ* composite film exhibits a low sheet resistance of 7.5 × 10^6^ ohm and high temperature coefficient of resistance of −2.79% K^−1^, close to the levels of the most important bolometric materials for uncooled infrared detectors. Particularly, a new low temperature reduction of graphene oxide induced by DAST, which is further enhanced by *in-situ* process, was discovered. This work presents valuable information about the DAST–graphene composite films, their chemical structures, mechanisms, physical properties, and comparison on *in-situ* and *ex-situ* syntheses of graphene–based composites, all of which will be helpful for not only theoretically studying the DAST and graphene materials and expanding their applications, but also for seeking new optoelectronic sensitive materials.

In the past decades, 4-*N*,*N*-dimethylamino-4′-*N* ′-methyl-stilbazolium tosylate (DAST) has attracted considerable attention^1^,^2^. Owing to its large nonlinear optical susceptibility and high electro-optic coefficient^1^,^2^, DAST has become one of the most important and successful organic nonlinear optical (NLO) materials that is applied widely in optical signal processing and frequency conversion^2^,^3^. Recently, new applications of DAST in terahertz (THz) generation and detection have also drawn great attention^4^. Therefore, DAST is an important and highly-attractive optical material. However, practical applications of DAST in optoelectronic or electronic devices have never been reported, largely due to its poor conductivity and the difficulty in preparing device-quality DAST–based thin films. Up until now, rather few literatures on DAST–based thin films have been published^5^,^6^.

On the other hand, graphene has also attracted tremendous research interest because of its unique structural features and outstanding electrical, optical, and mechanical properties^7^,^8^,^9^. Recently, it has been verified that composite films composed of graphene and metals or polymers exhibit excellent properties, so that they can be applied widely and efficiently in transistors^10^, supercapacitors^11^, mechanical springs^12^, photocatalysis^13^, and etc. Accordingly, we predicted that combination of DAST and graphene might provide a possibility to further improve the properties, by which new functional materials with exceptional properties might be developed. Moreover, we noted that both DAST and graphene are layered two-dimensional (2D) geometries^5^,^6^,^7^,^8^. Such structural similarity might be helpful for improving the integration of DAST with graphene, and thereby composite films with higher quality and performance might be produced. Therefore, study on the composite films that are composed of 2D DAST and 2D graphene would be intriguing from both fundamental and applied viewpoints. However, to the best of our knowledge, no relevant literature has been reported to date. Herein, we present the route to preparation of novel binary DAST–graphene composite films by addition of graphene during (*in-situ*) or after (*ex-situ*) DAST synthesis, as illustrated in Fig. 1. The chemical structures and physical properties of the products were systematically investigated and compared, by which the mechanisms and the differences between *in-situ* and *ex-situ* syntheses were deduced. Our experimental results will be helpful for not only promoting the theoretical research on *in-situ* and *ex-situ* syntheses of graphene–based composites^14^, but also for expanding the applications of DAST and inspiring studies on other materials.

## Results

The electrical properties of the as-prepared films were measured by a high resistance meter, the results of which are displayed in Fig. 2. It can be seen that the sheet resistance (*R*) at room temperature (*RT*) for a DAST film is very high, reaching 9.5 × 10^11^ Ω. When 5 wt% graphene is added after DAST synthesis to produce an *ex-situ* DAST–5% graphene composite film, *R*_*RT*_ is reduced slightly to 1.1 × 10^10^ Ω. However, when 5 wt% graphene is added during DAST synthesis to prepare an *in-situ* DAST–5% graphene composite film, *R*_*RT*_ is dropped dramatically to 7.5 × 10^6 ^Ω, a drop which is five orders of magnitude compared with that of the DAST film (Fig. 2). Figure 2 also shows that *R* of DAST increases with the temperature, suggesting a positive temperature coefficient of resistance (*TCR*, defined as 
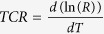
)^5^,^15^ for this material. In contrast, *R* for both *in-situ* and *ex-situ* composite films decrease with the elevated temperature, implying negative *TCR* for these composites. Further calculations reveal that the *TCR* values for the DAST film, *ex-situ* composite film, *in-situ* composite film, and graphene film are +1.45% K^−1^, −1.67% K^−1^, −2.79% K^−1^, and −4.43% K^−1^, respectively (Fig. 2). The positive *TCR* (+1.45% K^−1^) for the DAST film is due to the organic DAST^5^, while the negative *TCR* values of −1.67% K^−1^ and −2.79% K^−1^ suggest semiconductor characteristic for the resulting composite films. It is worth noting that lower *R*_*RT*_ (7.5 × 10^6 ^Ω) and higher *TCR* (−2.79% K^−1^), which are favorable for optoelectronic applications and close to the levels (*R*_*RT*_ = 0.2 – 2 × 10^5 ^Ω, *TCR* = −2.2% K^−1^)^15^ of vanadium oxide films (the most important bolometric materials for uncooled infrared (IR) detectors^15^,^16^), were measured from the *in-situ* composite films, compared with those of the *ex-situ* composite films. Therefore, the electrical properties of DAST can be significantly and rationally improved by graphene addition, and particularly, the *in-situ* composite films exhibit optimal electrical properties, suggesting the potential for serving as bolometric materials for uncooled IR or THz detectors.

The optical properties of the resulting films were investigated by UV-vis spectroscopy. Figure 3 shows that the transmittance (*T*) decreases significantly after graphene addition. Calculations indicate that the average *T* in the wavelength range of 600–1100 nm for the DAST film, *ex-situ* composite film, *in-situ* composite film are 43%, 34%, and 26%, respectively. Dramatic decrease of *T* is seen for the *in-situ* composite film. Moreover, an absorption peak for DAST at 506 nm is observed in Fig. 3, and this peak position almost remains unchanged after *ex-situ* synthesis, implying weak interactions between DAST and graphene in this composite. However, a significant change in line shape is observed in the *in-situ* composite film (Fig. 3), revealing strong interactions between DAST and graphene in this case. The lower the transmittance, the higher the response of the material is to the incident light, thus suggesting that the *in-situ* composite films also exhibit optimal optical response, similar to the electrical properties (Fig. 2).

In order to better understand the products and their properties, systematical characterizations were carried out. First, the morphologies were imaged by scanning electron microscopy (SEM), and the typical images are displayed in Fig. 4. It is seen that crystalline DAST film has been formed on the Si substrate (Fig. 4a). But numerous small DAST crystals appear if 5 wt% graphene is added after DAST synthesis to yield an *ex-situ* composite film (Fig. 4b). The film morphology is different when 5 wt% graphene is added during DAST synthesis to produce an *in-situ* composite film (Fig. 4c). In this case, large-scale continuous composite film containing 2D sheet-like nanostructures, as some other graphene–based composites^8^,^17^, was created uniformly and compactly on the Si surface (Fig. 4c). Clearly, the film continuity, uniformity, and compactness have been improved by *in-situ* synthesis.

The microstructures of the products were further investigated by high-resolution transmission electron microscopy (HRTEM). Figure 5a is a typical HRTEM image for the DAST film, in which the clear lattice fringes verify the formation of crystalline DAST film, as implied by SEM (Fig. 4a). The interplanar spacings of the lattice planes in Fig. 5a were estimated to be 0.24 nm, 0.21 nm, and 0.19 nm, assigned to the (100), (−111), and (−212) planes of DAST, respectively. The top-right inset of Fig. 5a shows a selected-area electron diffraction (SAED) pattern of the DAST film, in which the diffraction rings again confirm the yield of crystalline DAST film with the respective interplanar spacings of 0.24 nm, 0.21 nm, and 0.19 nm. However, the microstructure of the *ex-situ* DAST–5% graphene composite film is distinctly different (Fig. 5b). In this case, a loose film containing independent graphene nanosheets, as indicated by the red arrows, surrounded by amorphous organic DAST, as indicated by the blue arrows, is observed. The fuzzy diffraction rings in the SAED of Fig. 5b reveal disordered DAST in the *ex-situ* composite film. Meanwhile, some regular diffraction dots, originated from the ordered graphitic lattices, are visible in the SAED of Fig. 5b, revealing crystalline graphene nanosheets existed in the composite^18^. According to the diffraction dots in the SAED of Fig. 5b, the interplanar spacings were estimated to be 0.32 nm and 0.20 nm, corresponding to the (100) and (110) planes of graphene^18^,^19^,^20^, respectively. A HRTEM image for the *in-situ* DAST–5% graphene composite film is displayed in Fig. 5c. Notably, good integration of DAST with graphene nanosheets is seen in this situation, and thereby a composite film with more compact and uniform structure was formed. According to the SAED pattern of Fig. 5c, the interplanar spacings were calculated to be 0.38 nm, 0.32 nm, 0.29 nm, and 0.20 nm, respectively. Surprisingly, some data (0.32 and 0.20 nm) of graphene obtained from Fig. 5c agree well with those estimated from Fig. 5b, but some others (0.38 and 0.29 nm) deviate evidently. This suggests distortions of the graphene nanostructures in the *in-situ* process, originated from strong chemical interactions between DAST and graphene components in the *in-situ* composite films. Such valuable HRTEM images (Fig. 5) are highly beneficial for understanding the DAST–based materials and their properties, and they are presented for the first time in this work.

The crystallinity of the as-yielded films was characterized by X-ray diffraction (XRD), which results are displayed in Fig. 6. Figure 6 indicates that two peaks at diffraction angles of ∼6.6° and ∼12.7° were detected from DAST and DAST–graphene composite films. The peaks at ∼6.6° and ∼12.7° have been assigned to the respective signals from the (−212) and (−111) crystal planes of DAST^5^, clearly verifying the yield of highly crystalline DAST–based films in this work, as suggested by SEM (Fig. 4a) and TEM images (Fig. 5a). In contrast, a broad peak at ∼10.3° that is ascribed to the (001) plane of graphene oxide (GO)^13^,^17^,^21^, and a sharp peak at ∼33.3° that is assigned to the (110) plane of graphene^22^, were detected from a graphene film. This suggests existence of both GO and graphene components in the agent utilized in this work, and thus some oxygen-containing functional groups, *e.g.* −COOH, −C=O, −C−OH^8^,^21^,^23^, exist on the edges of the graphene nanosheets. For the composite films, besides the ∼6.6° and ∼12.7° peaks for DAST, two sharp peaks at diffraction angles of ∼25.7° and ∼33.3°, assigned to the respective signals from the (002) and (110) crystal planes of graphene^13^,^17^,^21^,^22^, appear simultaneously (Fig. 6). These XRD results (Fig. 6) agree well with those deduced from HRTEM (Fig. 5), both of which demonstrate that the main components of the as-prepared composite films are indeed DAST and graphene. It is worth noting that after composite formation, the peak at ∼10.3° for GO disappears, but a new peak at ∼25.7° for the (002) plane diffraction peak of graphene appears and the intensity of the peak for the (110) plane of graphene at ∼33.3° increases (Fig. 6). This evidently reveals conversion of GO to graphene or reduced graphene oxide (rGO)^23^ after composite synthesis^24^,^25^. Interestingly, this GO reduction occurs at a low temperature of 80 °C, much lower than those (500–1200 °C) in conventional heating methods^22^,^23^,^26^. Figure 6 also shows that both intensities of ∼6.6° and ∼12.7° peaks for DAST detected from the *in-situ* composite film are weaker than those measured from the *ex-situ* one, indicating stronger interactions between DAST and graphene in the *in-situ* process, as suggested by TEM results (Fig. 5). Based on the famous Scherrer equation of 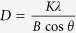
27 where *K* is the Scherrer constant (K = 0.89), *λ* is the wavelength of X-ray (0.154 nm), *θ* is the diffraction angle, and *B* is the full width at half maximum (FWHM) of a diffraction peak, the crystal size *D* can be estimated. Accordingly, the average *D* of DAST crystals were calculated to be 15.3 nm, 14.5 nm, and 13.5 nm for the DAST film, *ex-situ* composite film, and *in-situ* composite film, respectively. Smaller *D* in the *in-situ* composite film again reveals stronger interactions between DAST and graphene. But unexpectedly, both intensities of the peaks at ∼25.7° and ∼33.3° for graphene detected from the *in-situ* composite film are stronger than those measured from the *ex-situ* one (Fig. 6), which will be explained later in this work.

**Figure 1:**
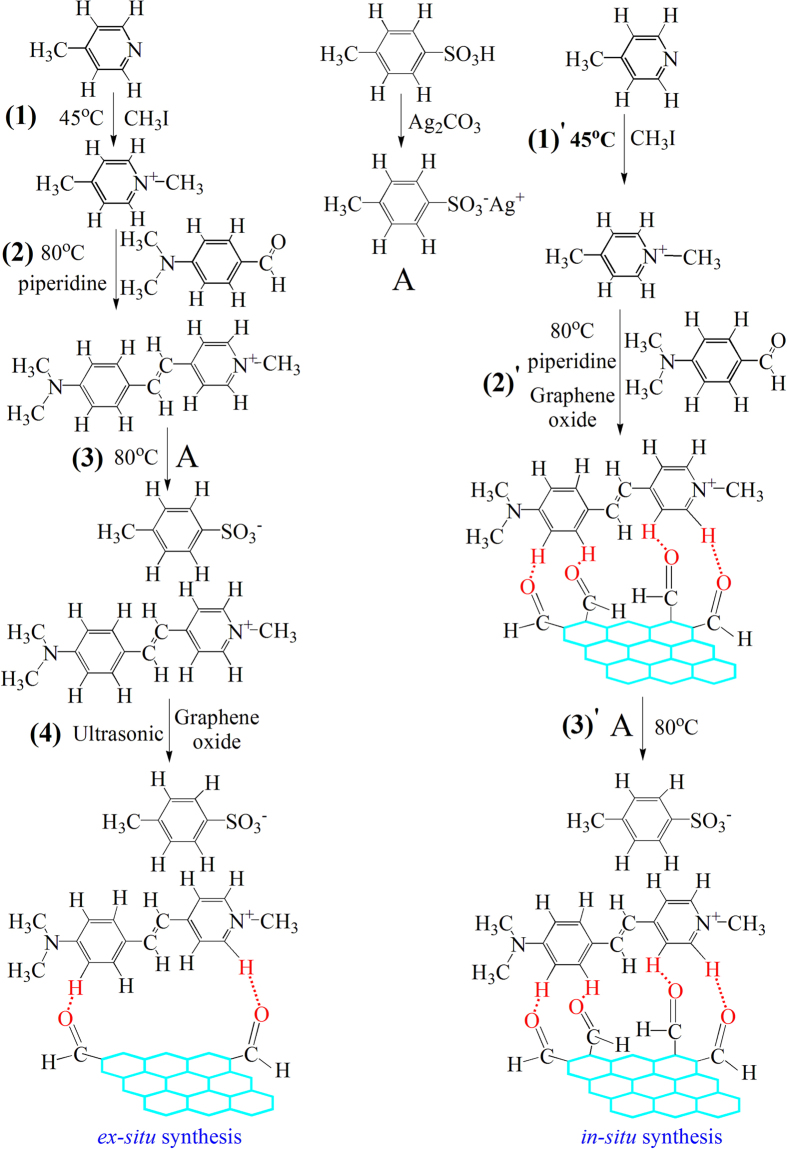
Schematic illustration of the processes for *ex-situ* (1-4) and *in-situ* (1-3)′ syntheses of DAST–graphene composite films.

Characterization of the products by IR spectroscopy provides further chemical information. Figure 7a shows the typical IR spectra at 4000–400 cm^−1^ wavenumber for various films. It is clear that *ex-situ* and *in-situ* DAST–graphene composite films exhibit similar IR features like those of DAST film (Fig. 7a), DAST crystal^4^,^28^, and DAST–CNT composite films^5^, again verifying basic DAST structures for the products in this work. Noticeably, a broad peak at ∼3242 cm^−1^, ascribed to the hydrogen (H) bonds of carboxyl (−COOH) groups of GO^29^,^30^, was clearly detected from the pristine graphene film, but this peak almost disappears after composite synthesis (Fig. 7a). Close inspection of IR spectra (Fig. 7b) reveals that compared with the DAST film, some peaks for the composite films, such as 1576 cm^−1^ (C=C vibrational mode)^28^, 1475 cm^−1^ (CH_3_ asymmetrical deformation mode)^28^, and 1158 cm^−1^ (ring C−H vibrational mode)^28^, shift to higher wavenumbers (blue-shifts) after graphene addition. Moreover, the blue-shifts of the peaks at 1576 cm^−1^ and 1158 cm^−1^ are similar levels (∼8 cm^−1^), but they are significantly larger than the shift (∼3 cm^−1^) of the peak at 1475 cm^−1^. Taking these together, we believe that new H bonds, different from those resulted from the carboxyl groups in GO, are created after composite synthesis, and they are mainly originated from the interactions between the H atoms in the rings of DAST and the O atoms in the C=O groups of GO. Notably, a peak at 1631 cm^−1^, assigned to C=C stretching of the sp^2^ character in GO^13^,^31^, was observed in a graphene film without mixing with DAST, but this peak disappears after composite synthesis (Fig. 7b). In contrast, a new peak at 1552 cm^−1^, reflected to the typical skeletal vibration of C=C in unoxidized graphene sheets^13^,^17^, was clearly detected from the *in-situ* composite (Fig. 7b), implying the restoration of the highly conjugated structure of graphene after chemical reduction^13^,^32^. These further demonstrate reduction of GO to graphene or rGO after composite synthesis, and particularly, more GO had been reduced to graphene in the *in-situ* process. As a result, stronger XRD signals for graphene were detected from the *in-situ* composite film (Fig. 6). It is worth noting that this GO reduction is induced by withdrawing^33^ of electrons from the H atoms in the rings of DAST to the O atoms in the C=O groups of GO, as illustrated in Fig. 1, markedly different from previous GO reductions^23^. In addition, larger blue-shifts (3–8 cm^−1^) are observed in the *in-situ* composite film than those (1–3 cm^−1^) in the *ex-situ* one (Fig. 7b), again indicating stronger interactions in the former, as suggested by TEM (Fig. 5) and XRD (Fig. 6) results. Therefore, IR results (Fig. 7) provide solid support not only for the strong chemical interactions between DAST and graphene in the *in-situ* composite, but also for the new reduction of GO at 80 °C induced by DAST. Moreover, Fig. 7 shows that the serial of IR transmittance is: *in-situ* composite film<DAST film<*ex-situ* composite film. This suggests that relatively compact film has been formed in the *in-situ* process, while relatively loose film in the *ex-situ* one, as revealed by SEM and HRTEM images (Figs 4 and 5) and resulted from stronger chemical interactions between DAST and graphene in the *in-situ* composite. Lower transmittance for the *in-situ* composite film (Fig. 7) suggests that such material also exhibits higher response to IR signals^5^, and thus greater potential for applications in IR detectors.

Raman spectroscopy is widely applied in characterizing the electronic structures of carbon products^23^,^34^,^35^,^36^,^37^. The typical Raman spectra for the resulting films are displayed in Fig. 8. Numerous characteristic Raman peaks of DAST were measured from the DAST film (Fig. 8), which are assigned as: C=C and C−C vibrational mode at 1587 cm^−1^; C=C in ring vibrational modes at 1620 cm^−1^, 1552 cm^−1^, 1437 cm^−1^, and 1322 cm^−1^; CH_3_ asymmetric deformation mode at 1480 cm^−1^; CH_3_ symmetric deformation mode at 1346 cm^−1^; ring C−H in-plane vibrational modes at 1212 cm^−1^, 1181 cm^−1^, and 1167 cm^−1^. These experimental results agree well with those theoretically simulated^28^, both of which are also in accordance with the IR measurements (Fig. 7). However, the Raman spectral features are completely different if DAST has been composed with graphene, in which new D band at ∼1327 cm^−1^ and G band at ∼1600 cm^−1^ were detected (Fig. 8). According to Fig. 8, the intensity ratios of the D and G bands (*I*_*D*_*/I*_*G*_) were calculated to be 0.80, 1.11, and 1.78 for the graphene film, *ex-situ* composite film, and *in-situ* composite film, respectively. Larger *I*_*D*_*/I*_*G*_ ratio might be attributed to two factors: one is the increase of defects in graphene, the other is higher structural disorder^37^. From Fig. 8, the full width at half maximum (FWHM) for G band were estimated to be ∼95 cm^−1^, ∼82 cm^−1^, and ∼74 cm^−1^ for the graphene film, *ex-situ* composite film, and *in-situ* composite film, respectively. Decrease of FWHM(G) suggests increase of the order of graphene^37^, which is further confirmed by stronger XRD signals for graphene after composite formation (Fig. 6). Thus, the structural factor on the *I*_*D*_*/I*_*G*_ ratio can be excluded in our case. Therefore, the increase of *I*_*D*_*/I*_*G*_ ratio in Fig. 8 reflects the increase of defects^37^, an indication of GO reduction^17^,^23^. Close inspection reveals that the G bands for the graphene film, *ex-situ* composite film, and *in-situ* composite film are located at 1600 cm^−1^, 1598 cm^−1^, and 1594 cm^−1^, respectively. Interestingly, red-shift of Raman G–peak is observed after composite synthesis (Fig. 8), opposite of the blue-shifts of IR spectra (Fig. 7b). Similarly, red-shift is seen in the D–peak. According to previous literatures^21^,^34^,^35^,^36^,^37^, binding energy shifts of Raman D and G bands for the composites in this work can be attributed to electron transfers to the graphene surfaces through additional chemical bonds between graphene and DAST. Particularly, more red-shifts for both G– and D–peaks are observed in the *in-situ* composite film compared with the *ex-situ* one (Fig. 8), again proving more chemical bonds created and electron transfers in the former. In fact, red-shifts of graphene signals in Raman spectra (Fig. 8) are exactly complementary with the blue-shifts of DAST signals in IR spectra (Fig. 7b). Despite of this, simultaneous observation of IR and Raman shifts has rarely been reported to date. Increase of *I*_*D*_*/I*_*G*_ ratio and red-shifts of G and D bands (Fig. 8) suggest interactions of GO with electron donator^35^,^36^, further verifying that our prediction of withdrawing^33^ of electrons from the H atoms in the rings of DAST to the O atoms in the C=O groups of GO (Fig. 1) is correct. Thus, IR and Raman measurements (Figs 7 and 8), coupled with the XRD results (Fig. 6), provide strong support for the low temperature reduction of GO induced by DAST. To the best of our knowledge, this is the first presentation about such low temperature reduction of GO and chemical interactions between DAST and graphene nanosheets.

## Discussion

**Figure 2:**
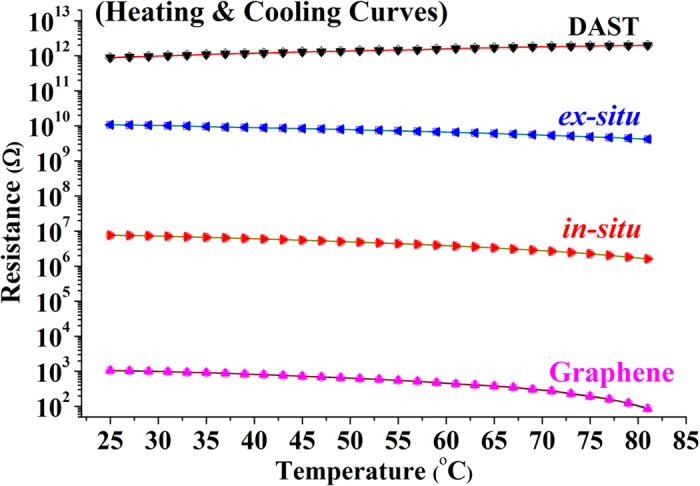
Electrical measurements of sheet resistances at different temperatures of DAST film, *ex-situ* composite film, *in-situ* composite film, and graphene film, respectively.

**Figure 3:**
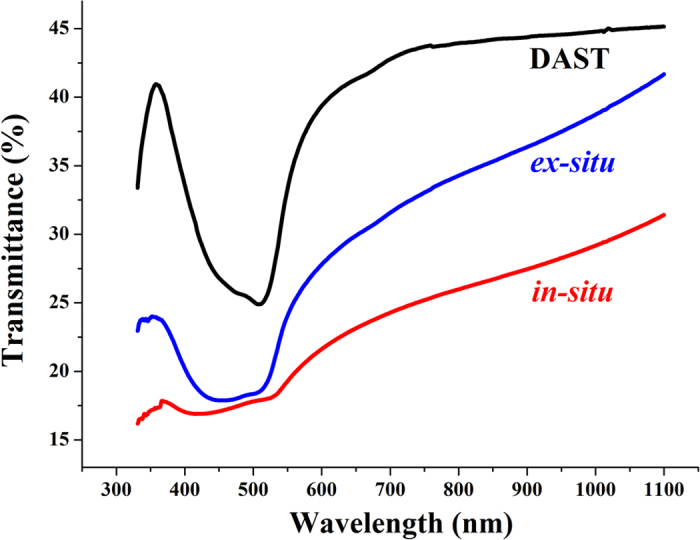
Typical UV-vis spectra of DAST film, *ex-situ* composite film, and *in-situ* composite film.

**Figure 4:**
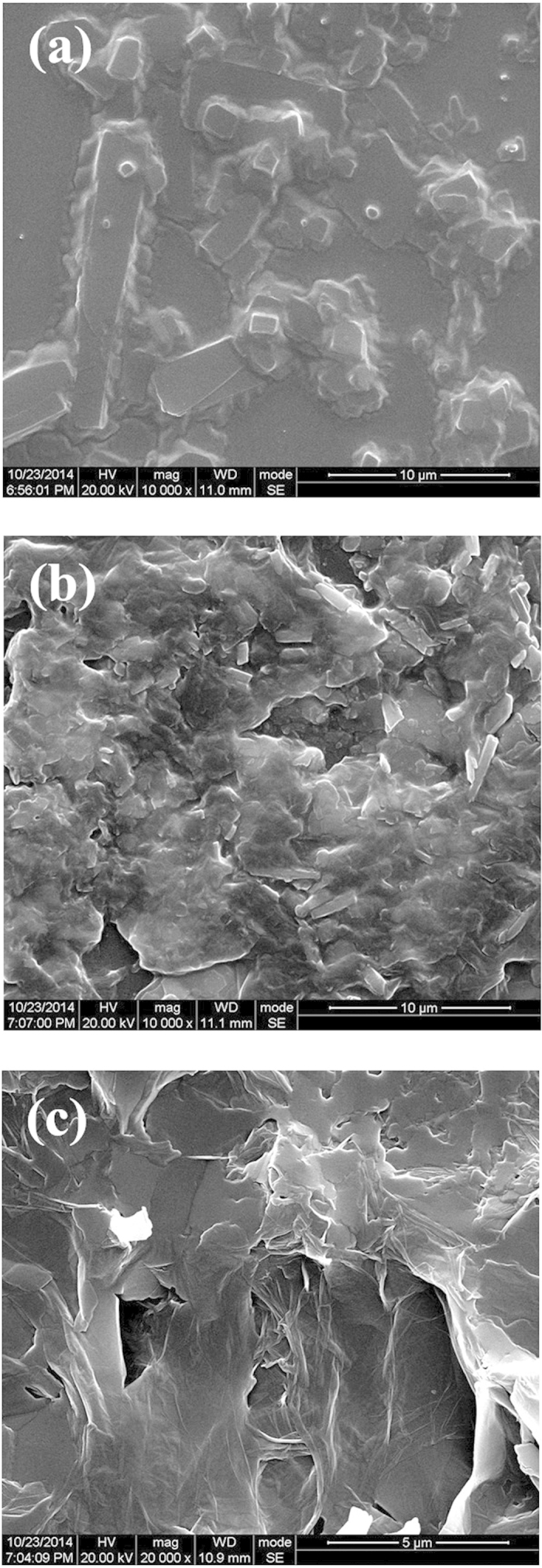
SEM images of (**a**) DAST film, (**b**) *ex-situ* composite film, and (**c**) *in-situ* composite film.

**Figure 5:**
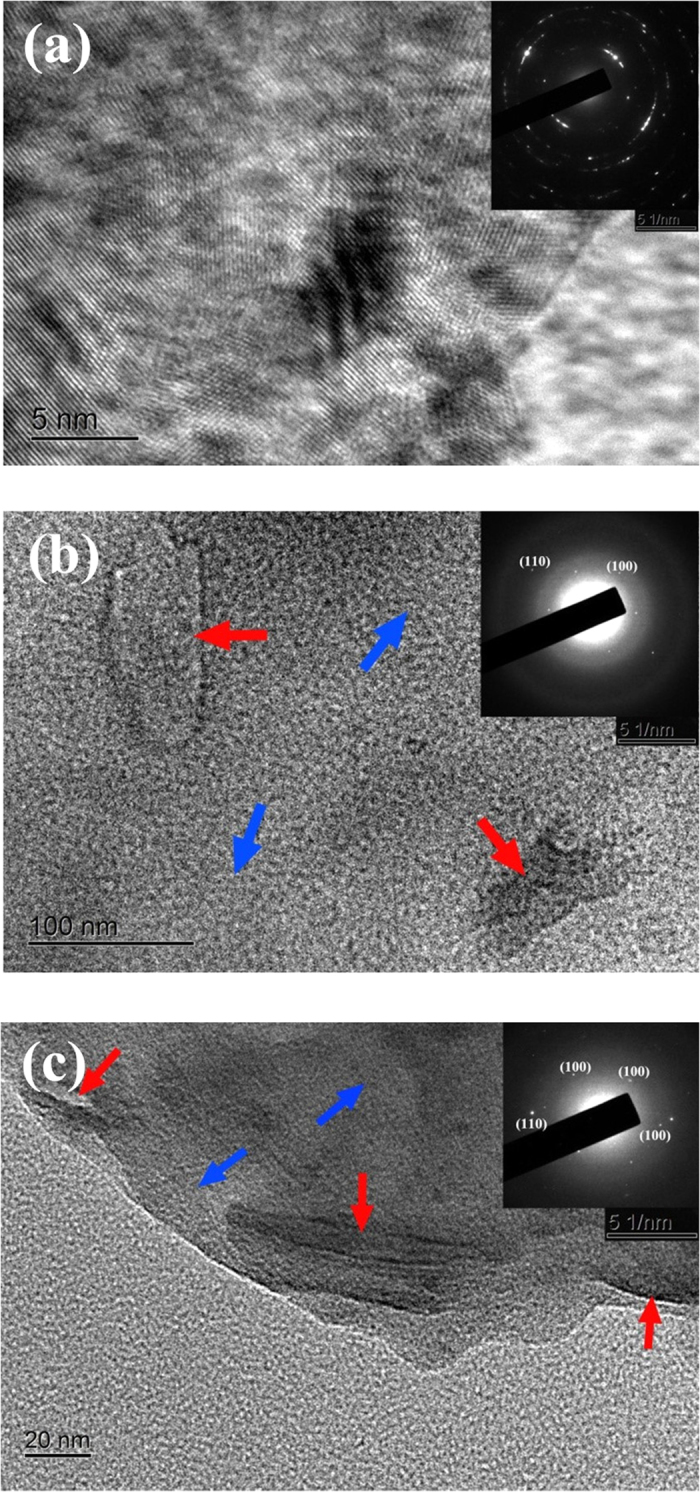
HRTEM images of (**a**) DAST film, (**b**) *ex-situ* composite film, and (**c**) *in-situ* composite film. The top-right insets are the electron-diffraction patterns.

**Figure 6:**
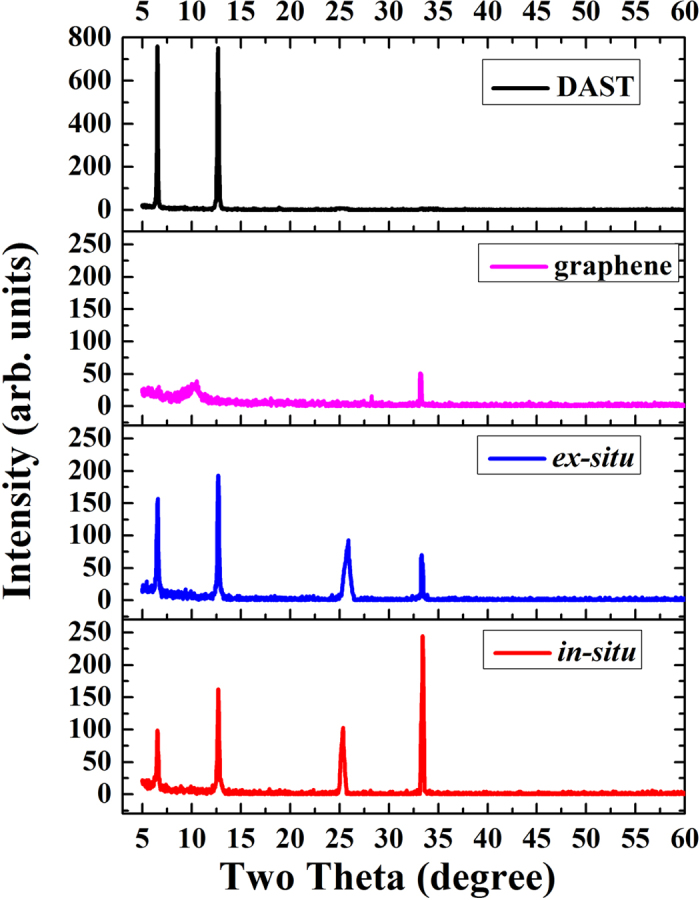
Typical XRD spectra of DAST film, graphene film, *ex-situ* composite film, and *in-situ* composite film, respectively.

**Figure 7:**
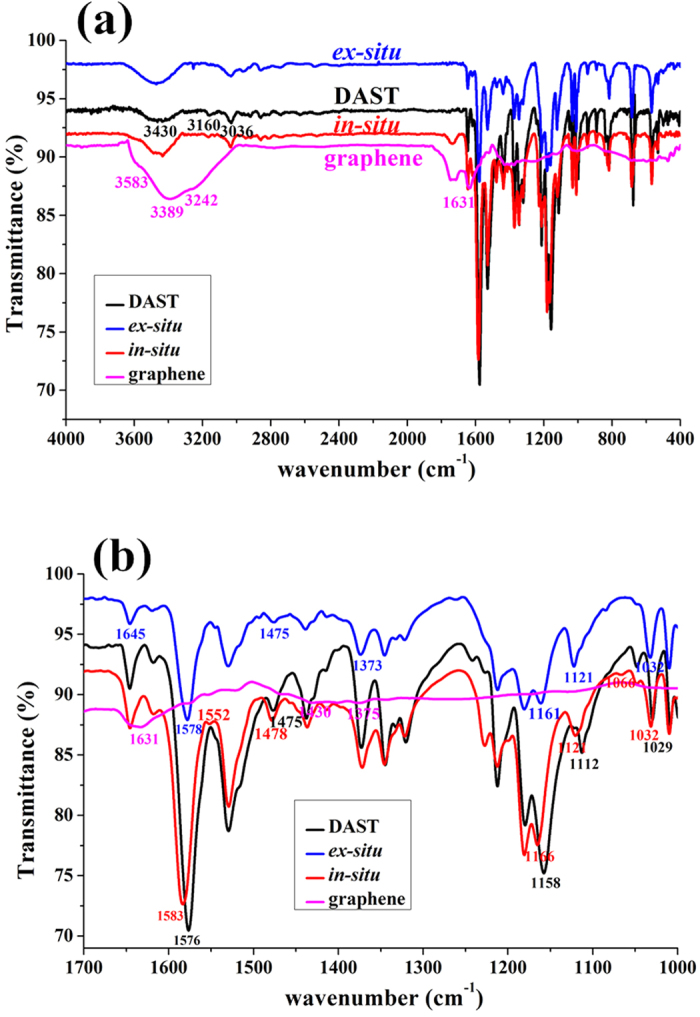
(**a**) Mid-IR spectra, and (**b**) comparison of IR spectra in 1000–1700 cm^−1^ for DAST film, *ex-situ* composite film, *in-situ* composite film, and graphene film.

**Figure 8:**
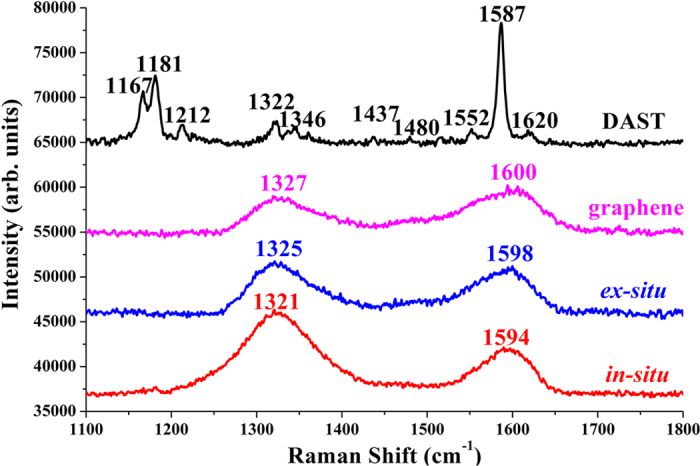
Typical Raman spectra of DAST film, graphene film, *ex-situ* composite film, and *in-situ* composite film, respectively.

**Figure 9:**
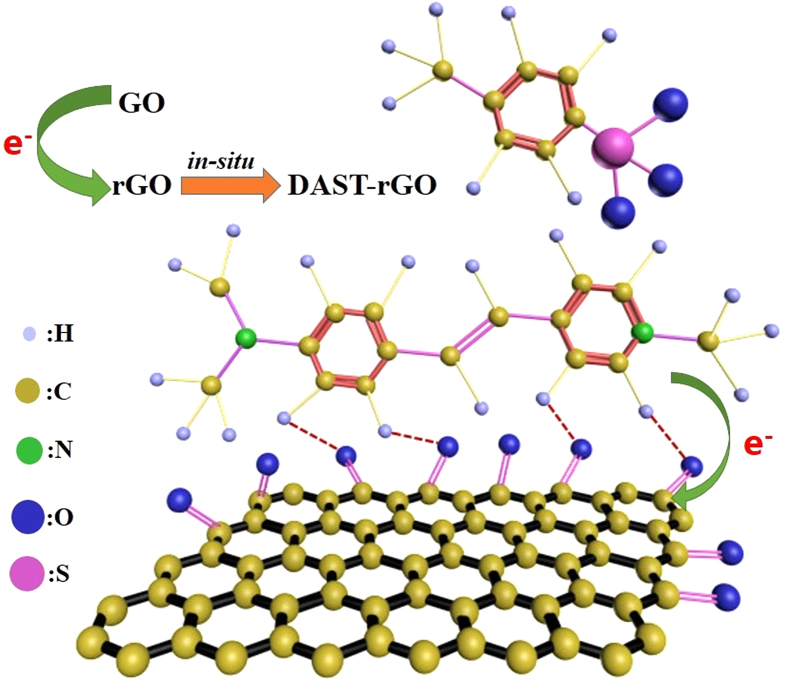
Schematic diagram showing the GO reduction induced by electron transfers from DAST molecules to graphene nanosheets through hydrogen bonds, and the *in-situ* synthesis of DAST–graphene composite.

Although the agents utilized are the same, the DAST–graphene composite films prepared by *in-situ* and *ex-situ* syntheses are significantly different (Figs 2–8). What is this attributed to? The answer can be traced from the structures and processes, as shown in Fig. 1. In an *in-situ* process, linear stilbazoliums ((CH_3_)_2_-N-C_6_H_4_-CH=CH-C_5_N^+^H_4_-CH_3_) are synthesized, and they are chemically attached on the surfaces of GO nanosheets through H bonds. This causes GO reduction and immediate (*in-situ*) formation of stilbazolium···rGO intermediates, which further react with tosylates (CH_3_-C_6_H_4_-SO_3_^−^) to produce the final DAST···rGO composite. The mechanism is further illustrated in Fig. 9. Strong H bonds result in intimate contacts of DAST with rGO, by which the products are compact and uniform, and thus the film quality, electrical conductivity, *TCR*, and optical response are significantly enhanced (Figs 2, 3, 4, 5, 6, 7). This suggests that the *in-situ* synthesis of DAST–graphene composite is mainly a chemical process, and the interface chemical engineering^30^ plays a key and positive role in this process. Notably, no agglomeration of rGO nanosheets is observed in the *in-situ* products; reversely, the resulting rGO nanosheets are dispersed uniformly in the DAST matrix (Figs 4c and 5c). This interesting phenomenon is attributed to that the agglomerations of rGO nanosheets have been efficiently suppressed by the strong chemical interactions between DAST and graphene. On the other hand, our results also suggest a new chemical functionalization of graphene by DAST. In this approach, GO nanosheets are reduced and *in*-*situ* functionalized by the DAST molecules (Fig. 9), in which no additional agents (e.g. stabilizers, surfactants, deoxidizers) are required, different from previous methods for functionalization of graphene^38^. With the excellent NLO^1^,^2^ and THz^4^,^5^ properties of DAST, the optical properties of graphene can be efficiently modified. However, in an *ex-situ* process, DAST has been pre-synthesized before graphene addition (Fig. 1). For a DAST molecule, the stilbazolium cation and tosylate anion are linked together with a deviation angle of 20°^39^. Thus, the spatial steric effects of big DAST molecules will negatively hinder the formation of H bonds between the pre-synthesized DAST and post-added GO. As a result, less H bonds are created, and loose films are yielded, which make the film quality, electrical conductivity, *TCR*, and optical response of the *ex-situ* composite films markedly weaker than those of the *in-situ* ones (Figs 2, 3, 4, 5, 6, 7). Therefore, the *ex-situ* synthesis of DAST–graphene composite is mainly a physical process, and the steric hindrance plays a critical and negative role in this process. Accordingly, one can deduce, as experimentally revealed in this work, that higher film quality and superior electrical and optical properties can be yielded by *in-situ* synthesis, compared with the *ex-situ* process. This suggests the first advantage of *in-situ* synthesis of graphene–based composites over *ex-situ* one, as previously reported^14^.

Moreover, our results indicate that a new reduction of GO is induced during DAST–graphene synthesis. How can GO be reduced? In general, GO can be reduced to graphene or rGO through methods of thermal annealing^25^, microwave irradiation^11^,^40^, photocatalyst^10^,^41^, chemical agent^42^, etc^23^. Although thermal annealing and solvothermal methods are applied widely in academic research, they are practically limited in device fabrications because of the negative effects of high temperature (500–1200)^23^ in these methods on other processes and sensitive materials, *e.g.* DAST will be decomposed at ∼260 °C^39^. In this situation, low temperature reduction of GO is favorable, which was previously achieved by microwave^40^ or photocatalyst^41^ method. Unfortunately, additional catalysts and microwave or photo irradiation are required in these methods^40^,^41^, and consequently, contamination of the products by the additional catalysts and/or chemical modification of the products by the additional microwave or photo irradiation will be inevitable. Although low temperature reduction of GO can also be achieved by addition of hydrazine or its derivatives agents^23^,^42^, this method is often challenged by the nocuous agents (hydrazine) and agglomerations of the resulting rGO nanosheets^23^. Rather different from all previous methods^23^, a new reduction of GO is induced by DAST in this work at a low temperature of 80 °C, much lower than those in previous methods^22^,^23^,^26^. Interestingly, this GO reduction is triggered by electron transfers through H bonds, namely withdrawing of electrons from the H atoms in the rings of DAST to the O atoms in the O=C of GO, as illustrated in Fig. 9. Although electrical conductivity can also be enhanced by *in-situ* polymerization of graphene–based composites^14^, condensation agents are required in the polymerization, and thereby contamination will also be unavoidable. Differently, it is DAST, one of the main components of the products, that promotes the GO reduction and formation of well-integrated composites in our approach. Therefore, our synthesis is a self-catalyzed process, in which no additional agents, catalysts, and/or irradiation are required, and thus, contamination of the products by the additional agents and/or chemical modification of the products by the additional treatments (microwave, photo, or ion irradiation) can be efficiently avoided. Moreover, the low temperature reduction of GO can be further enhanced by *in-situ* synthesis, due to stronger interactions between DAST and GO in this case. Low contamination and high-efficiency reaction are the second advantage of *in-situ* synthesis of graphene–based composites over *ex-situ* and previous others, which is unexpected and has never been pointed out previously.

Finally, we note that in the *in-situ* process, graphene nanosheets are integrated with DAST molecules through the O atoms in the O=C of GO with the H atoms in the rings of the stilbazoliums of DAST, as illustrated in Fig. 9 and revealed by SEM and TEM images (Figs 4c and 5c), which can be defined as a self-organized integration. However, in *ex-situ* process, graphene nanosheets are integrated with DAST molecules through the O atoms of GO randomly with the H atoms in the rings of stilbazoliums or those in tosylates of DAST, implying a random integration in this case (Figs 4b and 5b). Random integration will lead to weak interactions between graphene and DAST, and thus poor film quality and properties for the *ex-situ* products (Figs 2, 3, 4, 5, 6, 7). Based on the aforementioned experimental results, we believe that the *in-situ* DAST–graphene composite films hold great potential for practical applications in optoelectronic detectors. Imaginably, the applications of such intriguing materials would not be limited to these. According to Wan and co-workers^43^, the materials composed of porphyrin and graphene exhibit excellent solubility, dispersion stability, and optical limiting performance. Since DAST similarly contains ring structures and conjugated π–electrons like porphyrin, the DAST–graphene composite films might exhibit similar performances. With this possibility, the well-known NOL^1^,^2^ and THz^4^,^5^ properties of DAST, and the excellent electrical and optical properties presented in this work, one can predict that the DAST–graphene composite films could serve as multifunctional materials for both optical and optoelectronic applications, and particularly, better film quality and properties can be obtained by *in-situ* synthesis. Accordingly, self-organized integration and the resulting excellent multi-functions suggest the third advantage of *in-situ* synthesis of graphene–based composites over *ex-situ* one, which are also unforeseen and have never been noted previously.

In summary, we have successfully prepared novel DAST–graphene composite films by both *ex-situ* and *in-situ* syntheses. After addition of graphene to DAST, the film structures, electrical and optical properties can be dramatically modified, thus leading to conversion of DAST from a simple optical material to a versatile optoelectronic material. The interactions between DAST and graphene are strengthened in the *in-situ* process, but they are negatively suppressed by the steric hindrance of DAST molecules in the *ex-situ* process. Such strong interactions are utilized in the *in-situ* DAST–graphene composite films to enhance the GO reduction, integration of DAST with graphene, thus resulting in better film quality and higher electrical and optical properties. Therefore, the *in-situ* composite films exhibit comprehensive advantages for optoelectronic applications. Moreover, a new reduction of GO induced by DAST at a low temperature of 80 °C was discovered for the first time, which is essentially triggered by withdrawing of electrons from the H atoms in the rings of DAST to the O atoms in the C=O groups of GO. GO reductions induced by DAST and physical properties of DAST enhanced by graphene reveal the synergistic effects in the DAST–graphene composites. Valuable and systematic information presented in this work, including the novel DAST–graphene composite films, their chemical structures, physical properties, IR and Raman spectral features, self-catalyzed and self-organized integration, as well as a new low temperature reduction of GO, functionalization of GO by DAST, and comparison on *in-situ* and *ex-situ* syntheses of graphene–based composites, will be beneficial for further studies on the important DAST and graphene materials. Thus, we describe a facile and efficient way to convert the conventional optical materials to novel optoelectronic or electronic materials by addition of graphene, and thereby the applications can be greatly expanded. Particularly, the strategy presented here can be readily extended to studies on other composites, *e.g.* those composed of DAST derivatives or polymers and other 2D nanostructures^8^, one-dimensional (1D) nanowires or nanotubes^5^, zero-dimensional (0D) nanoparticles^13^,^17^, or even their combinations^12^, opening up new avenues for seeking novel multifunctional materials and controlling their optical and electrical properties for academic research and industrial applications.

## Methods

The preparation processes are illustrated in Fig. 1. Both *ex-situ* and *in-situ* syntheses were carried out at 80 °C. Details about the DAST–graphene composite syntheses, substrate pre-treatment, procedures for film deposition, and other related information are described in the “Supporting Information (SI)”. The as-prepared products were characterized by scanning electron microscopy (SEM, FEI INSPECT F), high-resolution transmission electron microscopy (HRTEM, JEOL JEM-2100F), high resistance meter (KEITHLEY 6517A), X-ray diffraction (XRD, Philips X’PertProMPD), Fourier infrared spectroscopy (PerkinElmer Spectrum 400), and micro-Raman spectroscopy (Renishaw, inVia), respectively.

## Additional Information

**How to cite this article**: Xu, X. *et al.* Conversion of 4-*N,N*-dimethylamino-4′-*N*′-methyl-stilbazolium tosylate (DAST) from a Simple Optical Material to a Versatile Optoelectronic Material. *Sci. Rep.* **5**, 12269; doi: 10.1038/srep12269 (2015).

## Supplementary Material

Supplementary Information
