# Supplementary material for: Conversion of 4-N,N-dimethylamino-4’-N’-methyl-stilbazolium tosylate (DAST) from a Simple Optical Material to a Versatile Optoelectronic Material
Source: Sci Rep. 2015 Jul 20;5:12269. doi: 10.1038/srep12269 (PMC4507482; doi:10.1038/srep12269)
Supplement: Supplementary Information [file srep12269-s1.pdf]

Conversion of 4-*N*,*N*-dimethylamino-4'-*N*'-methyl-stilbazolium tosylate (DAST) from a Simple Optical Material to a Versatile Optoelectronic Material

Xiangdong Xu[1,2]*, Ziqiang Sun[1], Kai Fan[1], Yadong Jiang[1,2], Rui Huang[1], Yuejiang Wen[1], Qiong He[1] & Tianhong Ao[1]

[1]*State Key Laboratory of Electronic Thin Films and Integrated Devices, Ministry of Education Key Laboratory of Photoelectric Detection & Sensor Integration Technology, School of Optoelectronic Information, University of Electronic Science and Technology of China (UESTC), Chengdu 610054, P.R. China*

[2]*Cooperative Innovation Center of Terahertz Science, University of Electronic Science and Technology of China (UESTC), Chengdu 610054, P.R. China*

*Correspondence and requests for materials should be addressed to X.X. (xdxu@uestc.edu.cn).

Supporting Information for "Conversion of 4-*N*,*N*-dimethylamino-4'-*N*'-methyl-stilbazolium tosylate (DAST) from a Simple Optical Material to a Versatile Optoelectronic Material"

In this presentation, DAST–graphene composite films were prepared by *in-situ* and *ex-situ* syntheses. The related procedures are illustrated briefly in Figure 1 of the text, and now, more details about them are described.

1. Synthesis of intermediates:

(1.1) 9.3 ml (0.1 mol) CH_3I and 10 ml ethanol were added to a 500 mL three hole round-bottom flask. Under reflux, 9.7 mL (0.1 mol) picoline was dropped slowly (for ~40 min) into the flask;

(1.2) Mixture of (1.1) was reacted at 45 ^oC for 10 min. After the reactants had been cooled, light yellow crystals that are insoluble in ethanol were crystallized. Filtrating and collecting the light yellow crystals, washing the products with ethanol, and then drying them in an oven at 60 ^oC for 24 hours. Consequently, 16.14 g white powders that are picoline iodized salt were obtained;

(1.3) 4.25 g (0.025 mol) $AgNO_3$ and 1.728 g (0.0125 mol) K_2CO_3 were added into 15 mL distilled water under stirring. After pale yellow precipitates had sufficiently come out, they were filtrated and washed. Then, the products were added into 17.5 mL aqueous solution containing 4.3085 g (0.025 mol) p-toluene sulphonic acid;

(1.4) After the pale yellow precipitates in (1.3) had been completely dissolved, the mixture was filtrated. Consequently, a clear solution was yielded;

(1.5) The clear solution (1.4) was evaporated in oil bath to obtain white solids. Then, the resulting white solids were dried in an oven at 60 ^oC for 48 hours, and thereby white powders that are silver p-toluene sulfonate (tosylate) were yielded.

(1.6) 1.49 g (0.01 mol) 4-N,N-dimethylaminobenzaldehyde and 1.882 g (0.01 mol) picoline iodized salt obtained in (1.2) were added to 25 mL ethanol, and the mixture was stirred. Then, the resulting solution (stilbazolium) was divided into two parts: One is A, another is B.

2. *in-situ* synthesis of DAST–graphene composite

(2.1) 0.16 g graphene was added into the A solution that had been obtained in (1.6), and then, the mixture was stirred and ultrasonically treated for 3 hours. Three drops of piperidine that play a role of catalyst were added slowly into the mixture under stirring. After reaction at 80 ^oC for 10 hours, the solution turned red.

(2.2) 2.793 g (0.01 mol) silver p-toluene sulfonate prepared in (1.5) was added into the solution (2.1), and then, the mixture was reflowed at 80 ^oC for 4 hours. After the reactants had been cooled down to room temperature, yellow precipitates and a red solution were yielded. Then, the mixture was ultrasonically treated at 40 ^oC for 4 hours, and the supernatant was reserved. After the supernatant had been evaporated, kermesinus powders were obtained. The resulting kermesinus powders are *in-situ* synthesized DAST–graphene composite.

3. DAST synthesis

(3.1) Three drops of piperidine that play a role of catalyst were added into the B solution obtained in (1.6) at 80 ^oC under stirring. After reaction for 10 hours, the

solution turned red.

(3.2) 2.793 g (0.01 mol) silver p-toluene sulfonate prepared in (1.5) was added into the mixture (3.1), and the mixture was reflowed at 80 ^{o}C for 4 hours. After the reactants had been cooled down to room temperature, yellow precipitates and a red solution were yielded. Subsequently, the mixture was filtrated, and the filtered solution was evaporated, by which red powders were obtained. Finally, the products were purified by recrystallization in methanol. The as-yielded red powders are DAST.

4. *ex-situ* syhthesis of DAST–graphene composite

(4.1) The DAST products prepared in (3.2) were divided into two parts, namely C and D;

(4.2) C of DAST obtained in (4.1) and 0.08 g graphene were added into 20 mL methanol. The mixture was ultrasonically treated at 40 ^{o}C for 24 hours;

(4.3) After the mixture (4.2) had been evaporated, light red powders were yielded, which are *ex-situ* synthesized DAST–graphene composite.

5. Preparation of *in-situ* DAST–graphene composite films

(5.1) 40 mg *in-situ* composite that was obtained in (2.2) was added into 5 mL methanol, and then, the solution was stirred and ultrasonically treated at 60 ^{o}C for 4 hours. Consequently, the resulting solution was dropped on a substrate that had been pre-treated (as shown in 8), and was spun for 1 minute (first low-speed spin coating at 500 r/min for 10 seconds, and then high-speed spin coating at 3000 r/min for 50 seconds).

(5.2) Repeating the process (5.1) for five times, and thus, *in-situ* DAST–graphene composite films were prepared.

6. Preparation of DAST films

(6.1) 40 mg DAST that was obtained in (3.2) was added into 5 mL methanol, and then, the solution was stirred and ultrasonically treated at 60 ^{o}C for 4 hours. Consequently, the resulting solution was dropped on a substrate that had been pre-treated (as shown in 8), and was spun for 1 min (first low-speed spin coating at 500 r/min for 10 seconds, and then high-speed spin coating at 3000 r/min for 50 seconds).

(6.2) Repeating the process (6.1) for five times, and thus, DAST films were prepared.

7. Preparation of *ex-situ* DAST–graphene composite films

(7.1) 40 mg *ex-situ* composite that was obtained in (4.3) was added into 5 ml methanol, and then, the solution was stirred and ultrasonically treated at 60 ^{o}C for 4 hours. Consequently, the resulting solution was dropped on a substrate that had been pre-treated (as shown in 8), and was spun for 1 min (first low-speed spin coating at 500 r/min for 10 seconds, and then high-speed spin coating at 3000 r/min for 50 seconds).

(7.2) Repeating the process (7.1) for five times, and thus, *ex-situ* DAST–graphene composite films were prepared.

8. Substrate pre-treatment

Si(100) wafers and glass were utilized as the substrate for film deposition. Before film preparation, the substrates were pre-treated through following processes:

(8.1) Ultrasonic baths in acetone and methanol for 20 min, respectively;

(8.2) Ultrasonic bath for 15 min in a mixture of concentrated H_2SO_4 and hydrogen peroxide solution with a ratio of 3:1;

(8.3) Chemical clean at 70 ^{o}C for 1 hour in a mixture of deionized water, ammonia, and hydrogen peroxide solution with a ratio of 5:1:1;

(8.4) The substrates were rinsed with deionized water;

(8.5) Finally, the as-cleaned substrates were dried by N_2 gas, after then, they were used in film preparation.

9. Characterizations

The as-prepared thin films were characterized by scanning electron microscopy (SEM, FEI INSPECT F), high-resolution transmission electron microscopy (HRTEM, JEOL JEM-2100F), high resistance meter (KEITHLEY 6517A), X-ray diffraction (XRD, Philips X'PertProMPD), Fourier infrared spectroscopy (PerkinElmer Spectrum 400), and micro-Raman spectroscopy (Renishaw, inVia), respectively.
